# A peer-facilitated psychological group intervention for perinatal women living with HIV and depression in Tanzania-Healthy Options: A cluster-randomized controlled trial

**DOI:** 10.1371/journal.pmed.1004112

**Published:** 2022-12-13

**Authors:** Sylvia Kaaya, Hellen Siril, Mary C. Smith Fawzi, Zenaice Aloyce, Ricardo Araya, Anna Kaale, Muhummed Nadeem Kasmani, Amina Komba, Anna Minja, Angelina Mwimba, Fileuka Ngakongwa, Magreat Somba, Christopher R. Sudfeld, Elysia Larson

**Affiliations:** 1 Department of Psychiatry and Mental Health, School of Medicine, Muhimbili University of Health and Allied Sciences, Dar es Salaam, Tanzania; 2 Management and Development for Health, Dar es Salaam, Tanzania; 3 Department of Global Health and Social Medicine, Harvard Medical School, Boston, Massachusetts, United States of America; 4 Centre for Global Mental Health, King’s College London, United Kingdom; 5 Department of Global Health and Population, Harvard T.H. Chan School of Public Health, Boston, Massachusetts, United States of America; 6 Department of Obstetrics and Gynecology, Beth Israel Deaconess Medical Center, Boston, Massachusetts, United States of America; 7 Obstetrics, Gynecology, and Reproductive Biology, Harvard Medical School, Boston, Massachusetts, United States of America; Stellenbosch University, SOUTH AFRICA

## Abstract

**Background:**

Perinatal women living with HIV (PWLH) have a greater risk of depression compared to other women; however, there are limited specialized mental health services available to them. We aimed to determine whether a stepped-care intervention facilitated by trained lay providers can improve mental health outcomes postpartum for PWLH.

**Methods and findings:**

Healthy Options is a cluster-randomized controlled study conducted in 16 government-managed antenatal care clinics that provided HIV care for pregnant women in urban Tanzania. Recruitment occurred from May 2015 through April 2016, with the final round of data collection completed in October 2017. Participants included a consecutive sample of pregnant women under 30 weeks of gestation, living with HIV and depression, and attending the study clinics. Control sites received enhanced usual care for depression (EUDC). Intervention sites received EUDC plus the *Healthy Options* intervention, which includes prenatal group sessions of problem-solving therapy (PST) plus cognitive behavioral therapy (CBT) sessions for individuals showing depressive symptoms at 6 weeks postdelivery. We assessed depressive symptoms comparable to major depressive disorder (MDD) using the Patient Health Questionnaire-9 (PHQ-9) with a locally validated cutoff at 9 months and 6 weeks postpartum. The primary time point is 9 months postpartum. We examined differences in outcomes using an intent-to-treat analysis with a complete case approach, meaning those with data at the relevant time point were included in the analysis. We used generalized estimating equations accounting for clustering. Of 818 women screened using the PHQ-9, 742 were determined eligible and enrolled (395 intervention; 347 control); 649 women (87.5%) participated in the first follow-up and 641 women (86.4%) in the second. A majority (270, 74.6%) of women in the intervention arm attended 5 or more PST sessions. Women enrolled in *Healthy Options* demonstrated a 67% (RR 0.33; 95% CI: 0.22, 0.51; *p*-value: <0.001; corresponding to a 25.7% difference in absolute risk) lower likelihood of depressive symptoms than women in control clusters at 6 weeks postpartum. At 9 months postpartum, women enrolled in *Healthy Options* demonstrated a nonsignificant 26% (RR 0.74; 95% CI: 0.42, 1.3; *p*-value: 0.281; corresponding to a 3.2% difference in absolute risk) lower likelihood of depressive symptoms than women in control clusters. Study limitations include not using diagnostic interviews to measure depression and not blinding data collectors to intervention status during follow-up.

**Conclusions:**

The Healthy Options intervention did not demonstrate reduction in depressive symptoms at 9 months postpartum, the primary outcome. Significant reductions were seen in depression symptoms at 6 weeks postpartum, the secondary outcome. Stepped-care interventions may be relevant for improving outcomes in the critical early postpartum window.

**Trial registration:**

Clinical Trial registration number (closed to new participants) NCT02039973

## Abstract 2

Elysia Larson and colleagues report the results of a cluster-randomized trial from Tanzania which investigates the impact of a peer-facilitated psychological intervention for perinatal women living with HIV and depression.

## Author summary

**Why was this study done?:**

- Depression during the pregnancy and postpartum periods is common, particularly among women living with HIV.
- In many resource-limited areas, availability of specialized mental health services is inadequate.
- Models of effective lay health worker-facilitated psychological interventions are needed to fill the care gap.

**What did the researchers do and find?:**

- A cluster-randomized controlled trial was conducted in 16 government-managed health clinics in urban Tanzania to examine the impact of a community-based health worker (CBHW)-led group intervention on symptoms of depression in the postpartum period.
- The stepped-care intervention included problem-solving therapy (PST) provided to all enrolled participants during pregnancy plus cognitive behavioral therapy (CBT) provided to participants who still showed symptoms of depression postpartum.
- At 9 months postpartum, the intervention group did not have a significant reduction in depressive symptoms compared to the enhanced control group. There was a 67% reduction in the likelihood of symptoms of depression at 6 weeks postpartum in the intervention group compared to the control group.

**What do these findings mean?:**

- The stepped-care model tested here was not effective in reducing symptoms of depression at 9 months postpartum, but was at 6 weeks postpartum.
- Additional interventions in the postpartum period need to be explored to significantly reduce symptoms of depression among pregnant women living with HIV in the longer term.

## Introduction

The prevalence of mental disorders, including major depressive disorder (MDD), is 2- to 4-fold higher in persons living with HIV than their HIV-negative counterparts [1,2]. Similarly, perinatal women living with HIV (PWLH) have more depressive symptoms than their HIV-negative counterparts during pregnancy and the postnatal period [3].

Depression in PWLH is associated with adverse HIV treatment and obstetric outcomes [4–6]. Ignoring depression within prevention of maternal-to-child transmission (PMTCT) services in low- and middle-income countries (LMICs) is a missed opportunity with significant short- and long-term consequences for the psychological and physical health of PWLH and their infants [7].

Contact coverage for current and recent clinically significant depression, defined as the proportion who sought professional help, was 23% in a population-based study in Ethiopia and Uganda, suggesting a large treatment gap [8]. This gap is largely driven by few mental health-specialized staff in health facilities [9] and few outpatient resources [10]. In Tanzania, integrated mental health services at primary and secondary levels of care are in their infancy, and specialized human resources for mental health services delivery are sparse [11].

Task-sharing interventions for PWLH and depression are a possible solution for psychological treatment of depression. Evidence from large randomized controlled trials demonstrates the effectiveness of interventions facilitated by trained lay providers or non-mental health-specialized health care providers for preventing or treating depressive disorders in primary care in both non-perinatal and perinatal women in LMIC [12,13], including pilot studies with samples from sub-Saharan Africa (SSA) [14,15]. However, not all interventions for perinatal depression in Africa have proven equally efficacious [16,17].

Few studies of SSA populations with moderate to severe levels of depression symptoms have evaluated the effects of lay health worker-facilitated psychological interventions on depression symptom severity in PWLH. This study aims to address this knowledge gap and reports on a large cluster-randomized controlled trial evaluating the effectiveness of a trained community-based health worker (CBHW)-facilitated psychological group intervention for reducing individual depressive symptoms among PWLH and depression in SSA.

## Methods

### Study setting and participants

We conducted a pair-matched cluster-randomized controlled trial within 16 study sites in 3 urban districts of Dar es Salaam, Tanzania: Ilala, Kinondoni, and Temeke. These 3 districts covered the entirety of Dar es Salaam at the time of recruitment in 2015 where HIV prevalence in women aged 15 to 19 years was 6.3% in 2016/2017 [18]. Within districts in Tanzania, there are hospitals at the secondary level of care and health centers and dispensaries at the primary level. Health centers are administratively and clinically linked to satellite dispensaries. We refer to the health center and its satellite facilities as “sites” or “clusters” and they make up the unit of randomization for this study.

Study sites were eligible for inclusion if they: (a) were government-managed clinics (dispensaries or health centers) providing integrated services for PMTCT; (b) were supported by a local non-governmental organization, Management and Development for Health (MDH); and (c) had at least 350 HIV-positive pregnant women registered in the year prior to study start. At the time of site selection, MDH supported 88% of otherwise eligible sites. We initially randomly selected 12 clusters from the list of those eligible. In November, the analysis team noted that there were fewer eligible women than expected in these clusters, so the study team identified, matched, and randomized 4 additional clusters, resulting in 16 total clusters [19]. Ten clusters consisted of a health center and a single satellite facility, and the remaining 6 sites had a single facility (no satellite facilities). Two additional changes were made to the initial selection. First, one of the primary control sites was upgraded from a health center to a hospital and was therefore not eligible as it would not have a reproductive and child health clinic. This clinic was moved to a nearby health center, so this new health center replaced the prior one as the study facility. Finally, in January 2016, one of the intervention facilities had fewer than expected women eligible so a new facility was randomly selected to replace that facility.

Women within selected study clusters were eligible for enrollment if they were: ≥18 years; at gestational age <30 weeks; were HIV-infected and receiving ART; demonstrated depressive symptoms on the Patient Health Questionnaire-9 (PHQ-9) comparable with MDD (score ≥9); had no current plan to harm themselves; and planned to continue postpartum care at the study facility. All eligible women were approached for participation.

### Randomization and masking

Clusters were stratified by district and pair-matched in each district using census classification of facility location as urban or peri-urban. Sites were then randomized to intervention or control arms with a 1:1 allocation ratio. Randomization within each pair occurred using a random number generator implemented by one of the coauthors. Facilities, patients, outcome assessment, and analysis were not blinded.

### Intervention

This study compared 2 arms: an intervention arm, delivering the “Healthy Options” intervention in addition to enhanced usual depression care (EUDC), and a control arm, which included only EUDC. The EUDC consisted of a 1-day training session for all study sites using an adaptation of the World Health Organization’s MhGAP depression treatment guidelines and focused on: (a) screening for depression using a locally validated version of the PHQ-9 [20,21]; (b) using algorithms for diagnostic assessment; and (c) depression management relevant to primary care settings, which included counseling to identify stressors and provide problem-solving support, offer information on sleep hygiene, facilitate reactivation of social support networks, and facilitate structured physical exercise. Providing follow-up care and knowing when to refer to specialized psychiatric care were also emphasized.

The Healthy Options intervention included evidence-based components from problem-solving therapy (PST) and cognitive behavioral therapy (CBT) implementable by trained lay facilitators in a stepped-care model. All individuals received PST during pregnancy and those still showing depressive symptoms postpartum received the CBT component. Licensed Tanzanian psychiatrists designed both the PST and CBT interventions. The PST was adapted from 2 manuals: the depression module of the World Health Organization’s MhGAP intervention guide for use in primary care [22] and the strengths-based “NAMWEZA” manual [23,24]. PST delivery strategies included social learning strategies in small group sessions to promote awareness of participants’ strengths, probe problems, build assertiveness, and practice effective communication skills using “I” statements. Participants articulated their future dreams and used the backlighting of dreams as a strategy for setting personal goals; feedback circles provided non-coercive opportunities to report using new skills. Session contents of the PST included acknowledging problems and identifying strategies for addressing them based on what has worked well in patients’ lives, exploring existing community support resources, and goal setting.

At the 6-week postdelivery survey, women screened for elevated depressive symptoms comparable with MDD using the PHQ-9 were invited to participate in 8 CBT group sessions. These sessions, adapted from the evidence-based “Thinking Healthy” intervention for psychosocial management of perinatal depression [25], were delivered as 8 once-weekly small group sessions. CBT content included: discussions of links between thoughts, feelings, and behavior; recognizing recurrent negative patterns of thinking that affect health; improving participant’s health and care for their infants to facilitate optimal growth and development; learning through role-playing; and sharing practical skills for addressing daily challenges and improving health and care for their infants. Encouragement to actively use new information and skills in between sessions was done by: facilitating discussions of more positive interpretations; sharing strategies used to manage stress and rekindle lost friendships and relationships; how to foster support from existing social networks; and sharing feedback on CBT homework assignments. The structured curricula used for these sessions is available on the author’s website.

Twenty-two CBHWs with prior counseling experience as ancillary workers provided HIV-related peer psychosocial support group counseling through facilitated group sessions. Their training involved a 1-week basic counseling skills refresher course, 2 weeks of intensive skills-building training for each part of the psychological intervention, and once-weekly for 4 weeks role-playing practice sessions. Five trained CBHWs were selected as supervisors based on performance and experience by their peers, and the study team led once-weekly, group, in-person supportive supervision sessions supported by 2 local psychiatrists. CBHW facilitated intervention group sessions with 15 to 20 women per PST groups (2 to 3 hours), along with 8 to 10 women per CBT session (1 to 1 ½ hours). Swahili was used to facilitate all sessions.

### Data collection

Data were collected using a research-assistant-administered survey, which took about 30 minutes to complete, and occurred at the health facility providing care. Private locations were used, proceeding with interviews once participants were comfortable and others would not overhear. During follow-up data collection, if unable to reach participants at the health facility, research assistants conducted interviews over the phone.

The 3 data collection time points were: baseline (May 2015 to April 2016), first follow-up, scheduled to be 6 weeks postdelivery (October 2015 to November 2016), and second follow-up, scheduled to be 9 months postdelivery (January 2016 to October 2017). We refer to the follow-up visits as 6 weeks and 9 months postpartum visits hereafter; however, it is important to note there is variability in the timing of each visit. Surveys at baseline and first follow-up were recorded on paper and entered into a Microsoft Access database by trained research assistants. Part-way through the first follow-up, we transitioned to a tablet-based data collection system using Open Data Kit-based software, which was also used for all of the second follow-up.

### Outcome measures

This study addressed 3 groups of outcome measures: the prespecified primary outcome, prespecified secondary outcomes, and other outcomes of interest. The primary outcome was depressive symptoms comparable to MDD as measured by the PHQ-9 at 9 months. The PHQ-9 score (range: 0 to 27) is our primary outcome, summarized as binary, using a locally validated cutoff of ≥9 for consistency with MDD [20].

We assessed secondary outcome measures at 9 months. They were: report of intimate partner violence (IPV) using items from the Domestic Violence Module of the Tanzania Demographic Health Survey [26], social support (measured on the Duke-UNC Functional Social Support scale from 1 to 4) [27], general self-efficacy scale (range: 1 to 4) [28], and HIV-related stigma (perceived and internalized stigma dimensions measured from 1 to 5) [29]. We report the average for each scale. Additional outcome measures include the primary and secondary measures measured at 6 weeks, as well as continuous PHQ-9 score measured both at 9 months and 6 weeks postpartum. All outcome measures were self-reported and assessed at the individual level.

### Statistical analyses

Power calculations for the primary binomial outcome of symptoms of depression comparable to MDD at 9 months postpartum were previously described in detail [19]. Briefly, the initial calculation assumed 12 clusters, each with a sample of 34 patients, stratified into 4 clusters per district and pair-matched. We assumed a coefficient of variation of 0.05, resulting in a minimum detectable risk ratio of 0.79. Given the final number of clusters was increased to 16, our power increased above 80% with alpha of 0.05.

In all analyses, participants were included in their assigned groups (intent-to-treat). For the primary outcome, we evaluated the relative risk of symptoms of depression comparable to MDD at 9 months postpartum between intervention and control groups using log-binomial generalized estimating equations with robust standard errors to account for clustering. We compared secondary outcomes at each time point between groups using log-binomial generalized estimating equations with robust standard errors to account for clustering for binary outcomes and generalized linear models with robust standard errors for continuous outcomes. All models included a fixed effect for district of the health facility to account for stratification by district in the randomization scheme. Although we present multiple outcomes, each analysis represents a distinct comparison and thus does not warrant adjustment for multiple comparisons [30].

We compared sociodemographic characteristics and outcome variables between the groups at baseline. As a sensitivity analysis, we conducted multivariable regression adjusting for hypothesized confounders, including age, marital status (married/living with partner versus other), completed secondary education, and employment (formal versus self-employment).

We conducted exploratory analyses to assess potential effect modification by the intervention on the primary outcome of symptoms of depression comparable to MDD at 9 months postpartum by baseline PHQ-9 score, report of IPV, social support, and HIV-related stigma. Statistical significance of effect modification was assessed with the Wald test.

For the primary outcome of interest, we assessed potential bias due to dependent censoring (missing outcome data) using inverse probability of censoring weights (IPCWs) [31]. We constructed stabilized censoring weights at each follow-up period including age, education, employment status, and marital status at baseline. For the final sensitivity analysis, we analyzed each outcome accounting for the pair-matched design using paired *t* tests in R software version 3.2. The IPCW analysis was conducted in SAS version 9.4 (SAS Institute, Cary, North Carolina, United States of America). All other statistical analyses were conducted using Stata version 14.2 (StataCorp LP, College Station, USA).

Ethical review boards at Harvard Medical School in the US and the National Institute for Medical Research in Tanzania approved the study. All participants provided written informed consent prior to participating and before randomization. The trial is registered at Clinicaltrials.gov (NCT02039973) and is closed to new participants. This study is reported as per the “CONSORT extension for Cluster Trials” guideline (see S1 CONSORT Checklist for details).

## Results

We enrolled 742 women from May 25, 2015 through April 29, 2016; 395 were in clinics randomized to receive the Healthy Options intervention and 347 in control clinics. The average cluster size was 46 people (range 10 to 98). Average age at baseline was 30 years. Most had completed at least primary school (86.7%) (Table 1). Women enrolled in the intervention and control facilities were similar on all demographic and household variables. Women in the intervention group were more likely to be married and less likely to have completed secondary school, but these differences were not statistically significant. Women were on average 11.1 weeks (median 9.3 weeks; range: 4 to 33.3) and 10.8 months (median 10.2 months; range: 8.0 to 23.5) postpartum at the first and second follow-up assessments, respectively.

**Table 1:** Baseline sociodemographic and outcome measures for study population.

		Control (*N* = 347) n (%)	Intervention (*N* = 395) n (%)
Demographic			
	Age, mean (SD)	29.5 (5.3)	29.8 (5.5)
	Married or living with partner	136 (39.5%)	190 (48.1%)
	Completed secondary school or higher	87 (25.2%)	71 (18.2%)
	Employed (formal or self-employed)	196 (56.6%)	200 (50.8%)
Outcome measures			
	Clinical symptoms comparable to MDD (PHQ-9 score ≥ 9)	317 (96.1)	361 (92.33)
	PHQ-9 score^a^	11.6 (3.0)	11.3 (3.2)
	Social support score^b^	3.0 (0.7)	2.9 (0.7)
	Self-efficacy score^b^	3.1 (0.6)	3.1 (0.8)
	HIV-related stigma score^a^	2.2 (0.7)	2.0 (0.7)
	Any IPV	53 (17.4%)	59 (16.3%)

^a^Lower values are better.

^b^Higher values are better.

IPV, intimate partner violence; MDD, major depressive disorder; PHQ-9, Patient Health Questionnaire-9; SD, standard deviation.

**Fig 1:**
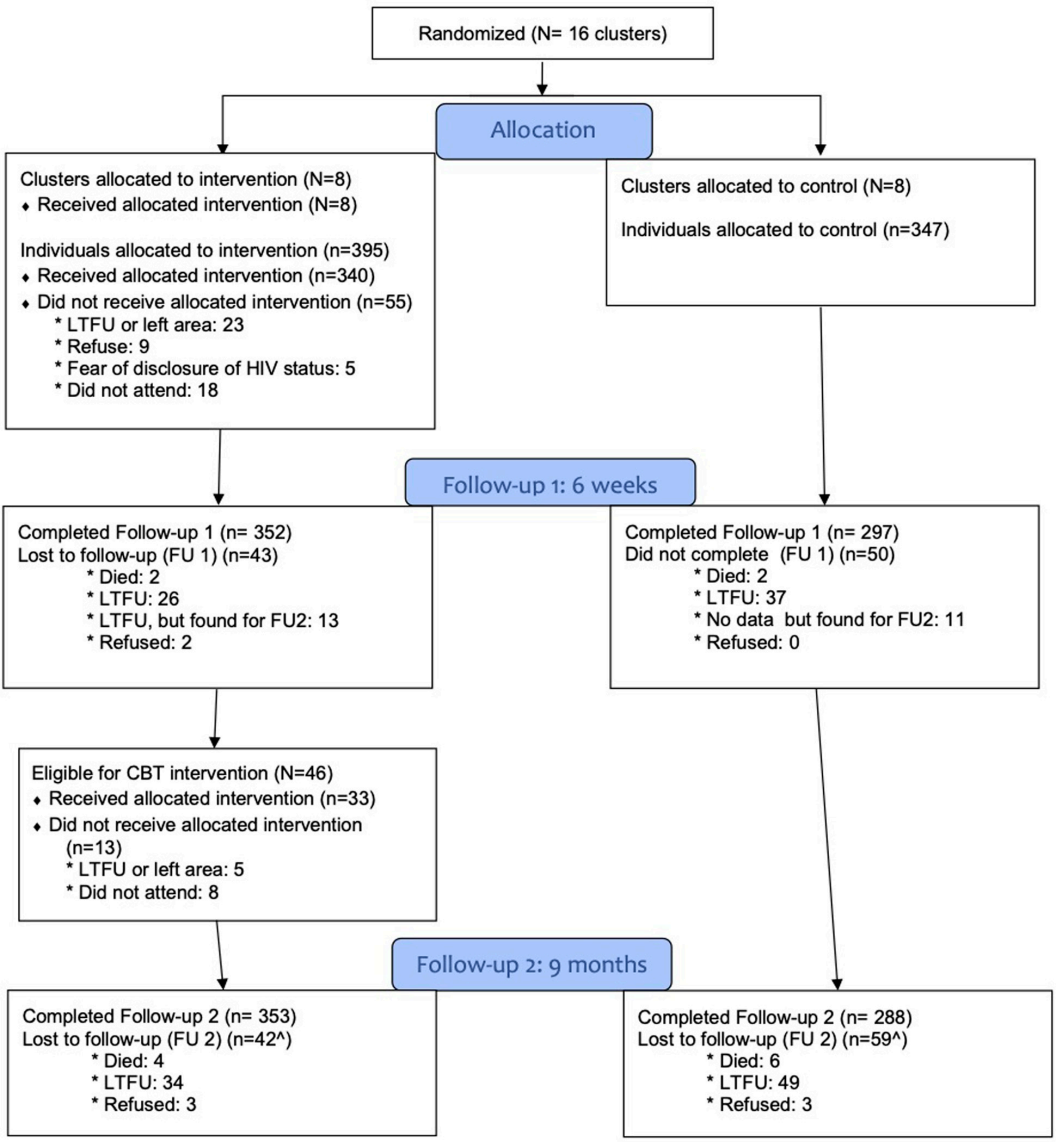
Participant flowchart.

Response rates were 87.5% (*n* = 649) at the 6-week follow-up (85.6% control, 89.1% intervention) and 86.4% at the 9-month follow-up (83.0% control, 89.4% intervention). The most common reasons for loss to follow-up were: moved out of the study area (*n* = 40, approximately 43%), participant unreachable after ≥3 attempts (*n* = 38, approximately 41%), death (*n* = 10, approximately 11%), and refusal (*n* = 5, approximately 5%) (Fig 1).

### Intervention participation

Most (93.9%) of the 362 women in the intervention arm attended at least 1 of the 7 predelivery structured PST sessions. More than one-third (37.3%) attended all 7 sessions and 74.6% attended ≥5 sessions. Of 341 women, 46 (13.5%) screened positive for symptoms of depression comparable to MDD at the first follow-up; 89.1% were reached and invited to attend a CBT intervention, with 8 additional structured once-weekly group sessions delivered by trained peer supervisors. Of these 41 women, most (75.6%) attended ≥7 sessions, and the remaining attended none (19.5%) or 1 session (4.9%). Information on participants who did not appear for any PST or CBT sessions was communicated to facility medical officers-in-charge, who ensured they were actively traced and encouraged to attend for facility-based assessment and referral for additional mental health care if needed.

### Intervention effects

The risk of depressive symptoms consistent with MDD in women in intervention clusters was 0.74 times (95% CI: 0.42, 1.3; *p*-value: 0.281) the risk in control clusters, at 9 months postpartum (Table 2) and 0.33 times (95% CI: 0.22, 0.51, *p*-value: <0.001) the risk in control clusters at 6 weeks postpartum (Table 3). The primary findings did not qualitatively change with the sensitivity analyses (S1 Appendix) including no meaningful difference in the effect of the intervention on risk of depressive symptoms consistent with MDD when using IPCW to account for non-response at follow-up: risk ratio of 0.33 (95% CI: 0.22, 0.50; *p*-value <0.001) at 6 weeks postpartum and risk ratio of 0.74 (95% CI: 0.41, 1.35; *p*-value: 0.33) at 9 months postpartum. The baseline differences between those who were retained in the sample and those who were lost to follow-up are presented in S3 Appendix. The PHQ-9 score on the continuous scale was 1.05 points lower in women in the intervention group compared to the control group (95% CI: 0.23, 1.87 lower; *p*-value: 0.012) at 9 months postpartum and 3.55 points lower (95% CI: 2.51, 4.59; *p*-value: <0.001) at 6 weeks postpartum. There was significant reduction in HIV-related stigma at both 9 months postpartum (0.24-point reduction; *p*-value: 0.012) and 6 weeks postpartum (0.40-point reduction; *p*-value: <0.001).

**Table 2:** Effect of the intervention on primary and secondary outcomes at second follow-up, approximately 9 months postpartum.

	Control mean (SD) or N (%)	Intervention mean (SD) or N (%)	Unadjusted^c^ mean difference or relative risk (95% CI)	Adjusted^d^ mean difference or relative risk (95% CI)
*Primary outcome*				
Clinical symptoms comparable to MDD (PHQ-9 score ≥ 9)	34 (12.0)	31 (8.9)	0.74 (0.42, 1.28)	0.71 (0.40, 1.25)
*Secondary outcomes*				
PHQ-9 score^a^	3.6 (3.4)	2.6 (3.7)	−1.05** (−1.87, −0.23)	−1.03** (−1.86, −0.19)
Social support score^b^	3.3 (0.6)	3.2 (0.7)	0.12 (−0.25, 0.48)	0.12 (−0.25, 0.48)
Self-efficacy score^b^	3.3 (0.6)	3.4 (0.7)	0.23* (−0.02, 0.48)	0.22* (−0.03, 0.47)
HIV-related stigma score^a^	1.7 (0.6)	1.5 (0.7)	−0.24** (−0.43, −0.05)	−0.25*** (−0.44, −0.06)
IPV				
Any	21 (10.4)	23 (8.8)	0.83 (0.53, 1.28)	0.84 (0.53, 1.34)
Sexual (any)	13 (6.5)	16 (6.1)	0.78 (0.53, 1.16)	0.84 (0.55, 1.27)
Physical (any)	12 (6.0)	11 (4.2)	0.70 (0.28, 1.74)	0.77 (0.32, 1.85)
Sexual and physical	4 (2.0)	4 (1.5)	0.73 (0.29, 1.88)	0.76 (0.27, 2.11)

^a^ Lower values are better.

^b^ Higher values are better.

^c^ The “unadjusted” model is adjusted for district of health facility to account for study design (stratification by district prior to randomization) as well as for clustering by health facility.

^d^ Adjusted for the following characteristics at baseline: age, married or living with partner, completed secondary education, and employed (formal or self-employment), district of health facility.

*** *p* < 0.01.

** *p* < 0.05.

* *p*<0.1.

CI, confidence interval; IPV, intimate partner violence; MDD, major depressive disorder; PHQ-9, Patient Health Questionnaire-9; SD, standard deviation.

**Table 3:** Exploratory analysis of the effect of the intervention on primary and secondary outcomes at first follow-up, planned 6 weeks postpartum.

	Control mean (SD) or N (%)	Intervention mean (SD) or N (%)	Primary unadjusted^c^ mean-difference or relative risk (95% CI)	Sensitivity analysis multivariable model^d^ mean-difference or relative risk (95% CI)
*Primary outcomes*				
Clinical symptoms comparable to MDD (PHQ-9 score ≥ 9)	111 (39.2%)	46 (13.5%)	0.33*** (0.22, 0.51)	0.32*** (0.22, 0.47)
*Secondary outcomes*				
PHQ-9 score^a^	6.9 (3.9)	3.4 (4.2)	−3.55*** (−4.59, −2.51)	−3.56*** (−4.55, −2.56)
Social support score^b^	3.4 (0.5)	3.2 (0.7)	0.05 (−0.27, 0.37)	0.05 (−0.26, −0.36)
Self-efficacy score^b^	3.3 (0.6)	3.3 (0.7)	0.21* (−0.03, 0.44)	0.20* (−0.03, −0.42)
HIV-related stigma score^a^	1.9 (0.5)	1.6 (0.6)	−0.40*** (−0.55, −0.26)	−0.41*** (−0.55, −0.26)
IPV				
Any	23 (9.7%)	28 (9.2%)	1.00 (0.72, 1.38)	0.96 (0.73, 1.27)
Sexual (any)	14 (5.9%)	17 (5.6%)	1.06 (0.95, 1.19)	0.93 (0.81, 1.07)
Physical (any)	18 (7.6%)	21 (6.9%)	0.93 (0.58, 1.49)	0.90 (0.57, 1.44)
Sexual and physical	9 (3.8%)	10 (3.3%)	0.86 (0.54, 1.37)	0.80 (0.57, 1.11)

^a^ Lower values are better.

^b^ Higher values are better.

^c^ The “unadjusted” model is adjusted for district of health facility to account for study design (stratification by district prior to randomization) as well as clustering by facility.

^d^ Adjusted for the following characteristics at baseline, which either showed imbalance at baseline or were identified as potential strong predictors of outcomes if imbalanced: age, married or living with partner, completed secondary education, and employed (formal or self-employment), district of health facility.

*** *p* < 0.01.

** *p* < 0.05.

* *p* < 0.1.

CI, confidence interval; IPV, intimate partner violence; MDD, major depressive disorder; PHQ-9, Patient Health Questionnaire-9; SD, standard deviation.

In exploratory analyses of effect modification, the intervention was associated with lower risk of symptoms of depression comparable to MDD at 6 weeks among women who did not report IPV at baseline compared to those who did (*p*-value for effect modification: 0.021), as well as among women who reported lower HIV stigma (lowest 2 tertiles) at baseline compared to those with higher HIV stigma (highest tertile) (*p*-value for effect modification: 0.006) (Table 4 and S2 Appendix). There were no adverse events associated with the intervention.

**Table 4:** Exploratory analysis of the effect of the intervention on symptoms of depression comparable to MDD at 6 weeks and 9 months postpartum stratified by effect modifiers.

		6-weeks postpartum		9 months postpartum	
		relative risk (95% CI)	*p*-value for effect modification	relative risk (95% CI)	*p*-value for effect modification
Baseline PHQ-9 score					
	Moderate (PHQ-9 score < 15)^a^	0.31 (0.20, 0.49)	0.216	0.86 (0.44, 1.65)	0.241
	Moderately severe or severe (PHQ-9 score ≥15)	0.47 (0.35, 0.63)		0.44 (0.16, 1.21)	
Report of IPV					
	Yes	0.53 (0.40, 0.71)	0.021	0.76 (0.49, 1.17)	0.796
	No	0.26 (0.14, 0.48)		0.64 (0.34, 1.22)	
Social support					
	Highest tertile of support	0.25 (0.13, 0.48)	0.517	0.46 (0.13, 1.59)	0.528
	Lowest 2 tertiles	0.34 (0.24, 0.49)		0.72 (0.43, 1.19)	
Hope					
	Highest tertile of hope	0.29 (0.12, 0.69)	0.405	0.63 (0.18, 2.23)	0.764
	Lowest 2 tertiles	0.38 (0.26, 0.54)		0.74 (0.48, 1.13)	
HIV-related stigma					
	Highest tertile of stigma	0.40 (0.29, 0.55)	0.006	0.49 (0.31, 0.78)	0.702
	Lowest 2 tertiles	0.23 (0.12, 0.47)		0.70 (0.26, 1.88)	

Baseline values used for each potential moderating factor; models used xtgee command in Stata version 14; Wald *p*-values reported.

^a^ Each level comparable to clinical symptoms of MDD. Levels consistent with previously validated cutoffs (Kroenke and colleagues (2001) and Fawzi and colleagues (2020)).

CI, confidence interval; IPV, intimate partner violence; MDD, major depressive disorder; PHQ-9, Patient Health Questionnaire-9; SD, standard deviation.

## Discussion

In this cluster-randomized trial, women in the intervention arm screened positive for clinically significant depressive symptoms at rates comparable to women in the EUDC arm at second (average 11 months postpartum) follow-up. However, they were significantly less likely to screen positive at first follow-up (average 11 weeks postpartum). Women randomized to peer-delivered PST and CBT group sessions had good adherence, with 74.6% attending ≥5 of 7 sessions, and 75.6% attending ≥7 of 8 sessions. This may suggest high acceptability of “talking therapies” delivered in a group format in the cultural context of the study.

Other studies also demonstrated positive effects of peer-led psychological interventions for depression in LMICs, including studies in South Africa [32], Zimbabwe [33], and Chile [12]. Our sample differs from these samples in including only PWLH and comorbid depression and having an EUDC rather than usual care as the control condition. Compared to similar studies from South Africa [16] and Nigeria [17], treatment adherence was better in our study and the intensity higher and duration longer of the psychological treatment. Our intervention effect size is similar to the modest pooled effect size of −0.61 (*p* = 0.06) from a meta-analysis evaluating psychological treatments for depression in people living with HIV in LMICs [34]. This meta-analysis demonstrated that depression outcomes did not vary by type of lead implementer (psychologist/counselor versus non-psychologist/lay counselor) or the delivery as an individual or group psychological intervention [34].

We identified 2 modifiers of the effect of the Healthy Options intervention on symptoms of depression comparable to MDD: The effects at first follow-up were not as strong among women reporting IPV or higher HIV-related stigma at baseline. This is important given evidence from the study context that IPV during the perinatal period is common—17% in our sample at baseline [19] and 19% among another sample of prenatal women in Dar es Salaam [35]. This suggests that interventions targeting IPV and HIV-related stigma be included in interventions for managing depression in PWLHA. This intervention did show a reduction in HIV-related stigma at both points of follow-up.

The intervention’s reduction in clinically significant depression systems compared to EUDC was only significant at the 6-week postnatal follow-up and not at the prespecified primary outcome time of 9 months. This is consistent with some [12] but not all studies [16,17,36]. As the intervention group showed considerable reductions in symptom severity at the 6-week visit, only 46 participants were eligible for the CBT component of the intervention. This means the possible added benefit of CBT at the group level would be low. In addition, the significant reduction in depression symptom severity in the control group may have had led to less statistical power to detect group differences at the 9-month period. A cluster RCT implementing a similar “Thinking Healthy” CBT program in Pakistan also found no significant reduction in symptoms at 6 months postpartum [37].

The decline in depression symptom severity in the control group over time, may have been due to expected declines in postpartum depression or to varying depression symptom severity over time. Implementing EUDC by providing psychoeducation and counseling may also have decreased symptoms in both intervention and control sites. Specific to this population, it is also possible that at the second follow-up (mean 11 months postpartum), women knew their infants’ HIV test results (which, while not testable in our study population, given the low rates of mother-to-child transmission in Dar es Salaam [38], were predominantly negative), which may have decreased stress and subsequently reduced depressive symptom severity in both groups. Particularly in resource-constrained settings, this evidence of a large reduction in depressive symptoms in the control group may suggest the need for better targeting of women with depression during the antenatal and early postpartum period when this intervention had the strongest effect.

Our study had several limitations. First, we did not use diagnostic interviews to measure depression due to the scarcity of mental health professionals. However, we used a locally validated Swahili version of the PHQ-9 to determine the cutoff for clinically significant depression against the gold standard of structured clinical assessments by mental health professionals in Tanzania [20]. Second, we were not able to blind data collectors to intervention status during follow-up data collection. While none of the data collectors were involved in intervention implementation, the potential for bias during face-to-face interviews exists. Third, the intervention was not compared to the current standard of depression care but rather to EUDC; intervention effects would likely be more pronounced if compared to usual care.

The trial findings demonstrate that symptoms of depression comparable to MDD were not reduced at 9 months postpartum using a stepped-care, peer-delivered, group PST and CBT intervention. However, the PST strategy was associated with reduced symptoms of depression comparable to MDD during the early postpartum period; given the critical period of cognitive development and physical growth in the first year of life [39], the positive effects in the early postpartum period can have significant long-term implications.

## Supporting information

S1 CONSORT Checklist CONSORT checklist for manuscript. (DOCX)

S1 Appendix Results of sensitivity analyses assessing *p*-values using different models. (DOCX)

S2 Appendix Effect of the intervention on secondary outcomes at 9 months postpartum stratified by potential effect modifiers. (DOCX)

S3 Appendix Baseline sociodemographic and outcome measures for women who had a 9-month postpartum follow-up PHQ-9 assessment as compared to women who did not have a follow-up PHQ-9 assessment. 3b: Baseline sociodemographic and outcome measures for women who had follow-up surveys as compared to women who did not have follow-up surveys.
(DOCX)

S1 Data De-identified data. (DTA)

## Abbreviations

CBHW: community-based health worker
CBT: cognitive behavioral therapy
EUDC: enhanced usual care for depression
IPV: intimate partner violence
IPCW: inverse probability of censoring weight
LMIC: low- and middle-income country
MDD: major depressive disorder
MDH: Management and Development for Health
PHQ-9: Patient Health Questionnaire-9
PMTCT: prevention of maternal-to-child transmission
PST: problem-solving therapy
PWLH: perinatal women living with HIV
SSA: sub-Saharan Africa
