# Supplementary material for: A peer-facilitated psychological group intervention for perinatal women living with HIV and depression in Tanzania-Healthy Options: A cluster-randomized controlled trial
Source: PLoS Med. 2022 Dec 13;19(12):e1004112. doi: 10.1371/journal.pmed.1004112 (PMC9746973; doi:10.1371/journal.pmed.1004112)
Supplement: S1 Appendix — (DOCX) [file pmed.1004112.s002.docx]

Appendix 1. Results of sensitivity analyses assessing p-values using different models

|  |  | Follow-up 1 |  |  | Follow-up 2 |  |
| --- | --- | --- | --- | --- | --- | --- |
|  | GEE p-value (corresponding to table 3) | Non-parametric p-value | Pair-matched p-value | GEE p-value (corresponding to table 2) | Non-parametric p-value | Pair-matched p-value |
| *Primary outcomes* |  |  |  |  |  |  |
| PHQ-9 score | <0.001 | <0.001 | 0.001 | 0.012 | 0.010 | 0.053 |
| Clinical symptoms comparable to major depressive disorder (PHQ-9 score ≥ 9) | <0.001 | - | 0.002 | 0.281 | - | 0.250 |
|  |  |  |  |  |  |  |
| *Secondary outcomes* |  |  |  |  |  |  |
| Social support score | 0.755 | 0.423 | 0.718 | 0.525 | 0.731 | 0.544 |
| Self-efficacy score | 0.086 | 0.586 | 0.171 | 0.072 | 0.356 | 0.113 |
| HIV-related stigma score | <0.001 | <0.001 | <0.001 | 0.011 | 0.009 | 0.028 |
| Intimate partner violence (IPV) |  |  |  |  |  |  |
| Any | 0.988 | - | 0.953 | 0.397 | - | 0.645 |
| Sexual (any) | 0.265 | - | 0.969 | 0.227 | - | 0.248 |
| Physical (any) | 0.758 | - | 0.860 | 0.446 | - | 0.713 |
| Sexual and physical | DNC | - | 0.889 | 0.519 | - | 0.781 |
