# Supplementary material for: A peer-facilitated psychological group intervention for perinatal women living with HIV and depression in Tanzania-Healthy Options: A cluster-randomized controlled trial
Source: PLoS Med. 2022 Dec 13;19(12):e1004112. doi: 10.1371/journal.pmed.1004112 (PMC9746973; doi:10.1371/journal.pmed.1004112)
Supplement: S2 Appendix — (DOCX) [file pmed.1004112.s003.docx]

Appendix 2. Effect of the intervention on secondary outcomes at 9 months postpartum stratified by potential effect modifiers

|  | Effect of intervention among participants that peported IPV at baseline  Mean difference [MD] (95% CI) or Relative risk [RR] (95% CI) | Effect of intervention among participants that did not report of IPV at baseline  Mean difference [MD] (95% CI) or Relative risk [RR] (95% CI) | p-value for effect modification | Effect of intervention among participants that peported higher stigma at baseline^a^  Mean difference [MD] (95% CI) or Relative risk [RR] (95% CI) | Effect of intervention among participants that reported lower stigma at baseline^b^  Mean difference [MD] (95% CI) or Relative risk [RR] (95% CI) | p-value for effect modification |
| --- | --- | --- | --- | --- | --- | --- |
| PHQ-9 score [MD] | -2.41 (-4.25, -0.56) | -3.75 (-5.02, -2.48) | 0.282 | -2.85 (-3.97, -1.72) | -3.89 (-4.99, -2.80) | 0.063 |
| Social support [MD] | -0.18 (-0.37, 0.00) | 0.04 (-0.30, 0.37) | 0.656 | -0.14 (-0.39, 0.12) | 0.04 (-0.30, 0.38) | 0.148 |
| Self-efficacy [MD] | 0.13 (-0.13, 0.40) | 0.20 (-0.05, 0.44) | 0.949 | 0.14 (-0.10, 0.37) | 0.23 (-0.05, 0.52) | 0.600 |
| HIV-related stigma [MD] | -0.11 (-0.33, 0.10) | -0.45 (-0.60, -0.31) | 0.001 | -0.17 (-0.40, 0.67) | -0.39 (-0.50, -0.29) | 0.114 |
