# Supplementary material for: A peer-facilitated psychological group intervention for perinatal women living with HIV and depression in Tanzania-Healthy Options: A cluster-randomized controlled trial
Source: PLoS Med. 2022 Dec 13;19(12):e1004112. doi: 10.1371/journal.pmed.1004112 (PMC9746973; doi:10.1371/journal.pmed.1004112)
Supplement: S3 Appendix — 3b: Baseline sociodemographic and outcome measures for women who had follow-up surveys as compared to women who did not have follow-up surveys. (DOCX) [file pmed.1004112.s004.docx]

Appendix 3a. Baseline socio-demographic and outcome measures for women who had a nine-month postpartum follow-up PHQ-9 assessment as compared to women who did not have a follow-up PHQ-9 assessment.

|  |  | **Had a PHQ-9 assessment (N=634)**  **n (%)** | **Did not have a PHQ-9 assessment (N=108)**  **n (%)** | **p-value** |
| --- | --- | --- | --- | --- |
| Demographic | |  |  |  |
|  | Age, mean (SD) | 29.7 (5.4) | 29.0 (5.4) | 0.415 |
|  | Married or living with partner | 471 (74.5%) | 65 (60.7%) | 0.085 |
|  | Completed secondary school or higher | 129 (20.5%) | 29 (26.9%) | 0.255 |
|  | Employed (formal or self-employed) | 332 (52.5%) | 64 (59.3%) | 0.181 |
| Outcome measures | |  |  |  |
|  | Clinical symptoms comparable to major depressive disorder (PHQ-9 score ≥ 9) | 579 (93.8) | 99 (95.2) | 0.001 |
|  | PHQ-9 score ^a^ | 11.3 (3.0) | 12.3 (3.4) | 0.002 |
|  | Social support score ^b^ | 3.0 (0.7) | 2.9 (0.7) | 0.287 |
|  | Self-efficacy score ^b^ | 3.1 (0.7) | 3.1 (0.7) | 0.845 |
|  | HIV-related stigma score ^a^ | 2.1 (0.7) | 2.2 (0.7) | 0.279 |
|  | Any Intimate partner violence (IPV) | 95 (16.5%) | 17 (18.7%) | 0.570 |

^a^ Lower values are better

^b^ Higher values are better

Appendix 3b. Baseline socio-demographic and outcome measures for women who had follow-up surveys as compared to women who did not have follow-up surveys.

|  |  | **Women with response at first follow-up (N=649)**  **n (%)** | **Women without response at first follow-up (N=93)**  **n (%)** | **p-value** | **Women with response at second follow-up (N=641)**  **n (%)** | **Women without response at second follow-up (N=101)**  **n (%)** | **p-value** |
| --- | --- | --- | --- | --- | --- | --- | --- |
| Demographic | |  |  |  |  |  |  |
|  | Age, mean (SD) | 29.7 (5.4) | 29.3 (5.3) | 0.488 | 29.8 (5.4) | 28.8 (5.3) | 0.288 |
|  | Married or living with partner | 475 (73.4%) | 61 (66.3%) | 0.050 | 476 (74.5%) | 60 (60.0%) | 0.040 |
|  | Completed secondary school or higher | 135 (21.0%) | 23 (24.7%) | 0.646 | 129 (20.3%) | 29 (28.7%) | 0.090 |
|  | Employed (formal or self-employed) | 345 (53.2%) | 51 (55.4%) | 0.439 | 334 (52.3%) | 62 (61.4%) | 0.056 |
| Outcome measures | |  |  |  |  |  |  |
|  | Clinical symptoms comparable to major depressive disorder (PHQ-9 score ≥ 9) | 598 (94.2) | 80 (93.0) | 0.614 | 586 (93.9) | 92 (94.8) | 0.037 |
|  | PHQ-9 score ^a^ | 11.4 (3.0) | 11.8 (3.5) | 0.099 | 11.3 (3.0) | 12.3 (3.5) | <0.001 |
|  | Social support score ^b^ | 3.0 (0.7) | 2.8 (0.7) | 0.162 | 3.0 (0.7) | 2.9 (0.7) | 0.566 |
|  | Self-efficacy score ^b^ | 3.1 (0.7) | 3.0 (0.7) | 0.121 | 3.1 (0.7) | 3.1 (0.7) | 0.837 |
|  | HIV-related stigma score ^a^ | 2.0 (0.7) | 2.2 (0.8) | 0.001 | 2.1 (0.7) | 2.2 (0.7) | 0.126 |
|  | Any Intimate partner violence (IPV) | 103 (17.2%) | 9 (12.9%) | 0.094 | 96 (16.5%) | 16 (18.8%) | 0.562 |
